# Reconstruction of Cavus Foot: A Review

**DOI:** 10.2174/1874325001711010651

**Published:** 2017-07-31

**Authors:** Bom Soo Kim

**Affiliations:** Department of Orthopaedic Surgery, Inha University College of Medicine 7-241, Sinheung-dong 3-ga, Jung-gu, Incheon 400-712, Republic of Korea

**Keywords:** Cavovarus, Cavus, Deformity, Osteotomy, Reconstruction, Subtle

## Abstract

Cavus foot ranges from flexible subtle to rigid severe deformities, and is related to many pathological conditions of the foot and ankle. Understanding the deformity and the deforming force is essential in treating the cavus foot as well as the associated comorbidities. Since every deformity is different, surgical plans should be customized to each patient.

## 1 INTRODUCTION

Cavus foot encompasses a wide range of deformities, from a subtle flexible deformity to severe rigid cavus foot. The prevalence of cavus foot is reported to be 10 to 25% of the population or as common as flat foot [1-3]. With increasing awareness of the milder or subtle cavus, the whole disease entity may be more prevalent.

Cavus foot is related to many pathological conditions of the foot and ankle. Even a subtle structural deformity or muscle imbalance can create repeated problems. However, cavus foot seldom draws attention unless the deformity is very severe. Understanding the underlying deformity and eliminating the deforming force is essential to successful treatment. In this article, the clinical and radiological diagnosis of the cavus foot and the state-of-the-art surgical procedures have been reviewed.

### 2 ETIOLOGY

The term “pes cavus” or “cavus foot” is used to describe a wide spectrum of foot shapes that have an abnormal elevation of the medial longitudinal arch [4]. High arch of the foot is frequently associated with hindfoot varus, forefoot adduction and plantar flexion, and ankle equinus.

The etiology is most frequently attributed to the neuromuscular disorders involving brain, spinal cord, or the peripheral nerves. Two thirds of adults with symptomatic cavus foot have an underlying neurological condition [5]. Among them, Charcot-Marie-Tooth (CMT) disease, a hereditary sensory motor neuropathy, is most frequently reported. The probability of a patient who has bilateral cavovarus feet being diagnosed with CMT is 78% [6].

The progressive muscle involvement from distal to proximal, most frequently affects the intrinsic muscles, the tibialis anterior, and the peroneus brevis. Extensor hallucis longus is relatively spared. Relative weakness in one of the two opposing muscles causes muscle imbalance and structural deformity. Structural deformation is more substantial when the motor imbalance begins before maturation of the skeleton [4, 7].

Muscle imbalance can occur between the extrinsic and intrinsic muscles, between the posterior tibial and the peroneus brevis muscles, and between the anterior tibial and the peroneus longus muscles. Weak anterior tibial relative to the peroneus longus results in plantar flexion of the first metatarsal. The flexion power of the peroneus longus becomes much stronger as the foot is positioned in equinus [2, 8]. Recruitment of extensor hallucis longus produces cock up deformity of the great toe, which further depresses the metatarsal head. With weak intrinsic muscles, the unopposed extensor digitorum longus hyperextends the unstable lessor toes at the metatarsophalangeal joint while the flexor digitorum longus and brevis flex the phalanges. The resultant claw toe deformity and plantarflexed metatarsal heads amplify forefoot equinus [4, 7].

The plantar flexed forefoot forces the hindfoot into varus. Hindfoot varus is initially flexible, but can gradually become rigid over time. With the rigid hindfoot varus, the Achilles tendon becomes a secondary invertor and becomes contracted [4, 7].

A mild variation of the cavovarus deformity without an identifiable underlying neurological deficit has been increasingly reported in recent literature [2, 9, 10]. This foot shape has been referred to as the subtle, nonneurologic, or idiopathic cavus. Although the exact etiology for this entity has been subject to debate, both intrinsic and extrinsic muscle imbalances may play a role [11, 12]. Chronic ankle instability with a varus-tilted mortise can also result in a cavovarus foot.

### 3 CLINICAL MANIFESTATIONS

Cavus foot, even subtle deformity, can cause various problems through out the foot and ankle (Table **1**). Metatarsalgia due to forefoot overload is related to the combined effect of cavus foot and tight heel cord. When examining a patient with metatarsalgia, cavus foot should be in the list of differential diagnoses along with Morton’s neuroma and long metatarsals. Overload on the 1^st^ metatarsal head can lead to sesamoiditis or sesamoid fractures. Overload on the lateral border can result in stress fracture of the 5^th^ metatarsal. Stress fracture of the 5^th^ metatarsal is difficult to treat without addressing the underlying cavus deformity.

Reduced shock absorption due to rigid hindfoot and tight heel cord can lead to plantar fasciitis or Achilles tendinitis. Haglund deformity can become symptomatic more easily if the heel is in varus because the posterior superior calcaneal tuberosity will become more prominent. Rigid joints can progress to joint destruction and develop arthritis over time.

Chronic lateral ankle instability and recurrent sprain is inevitable in a patient with cavus foot. Prolonged lateral overload and recurrent sprain can lead to peroneal tendon problems. Any attempt to repair the lateral ligamentous problems will not be successful if the bony structure is remained in varus. If left untreated, prolonged cavus foot will eventually lead to varus ankle osteoarthritis.

### 4 PHYSICAL EXAMINATION

**Fig. (1):**
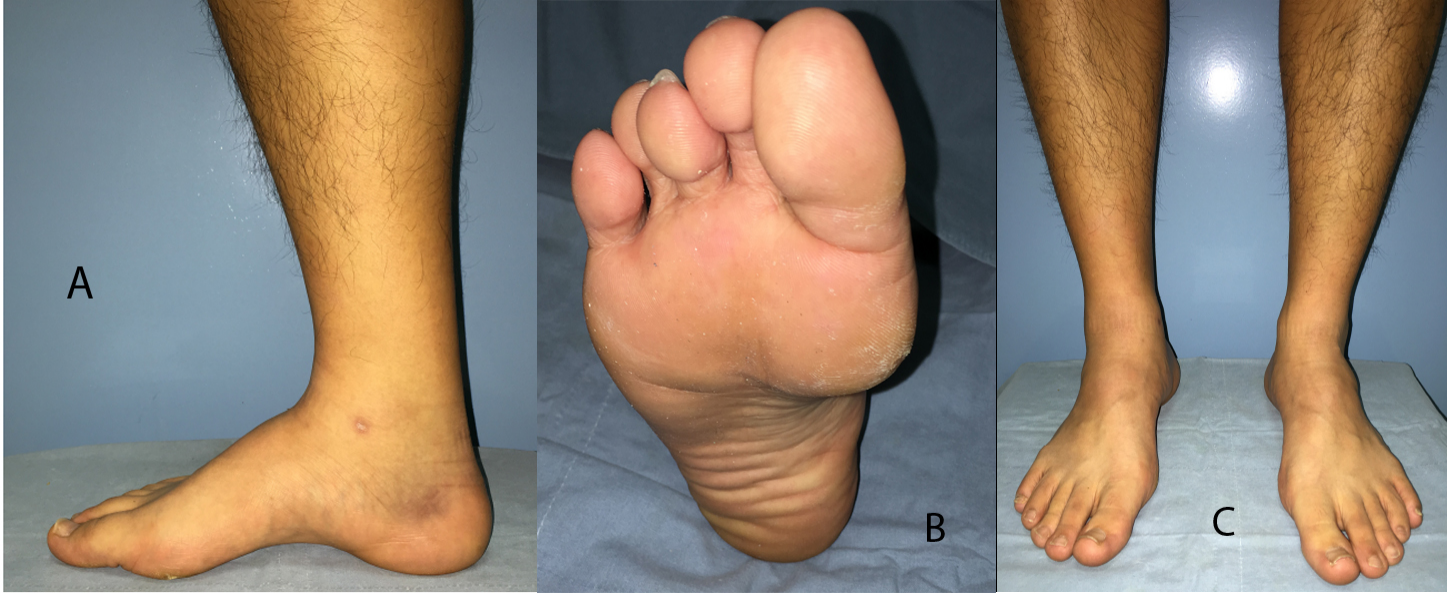
A 19 year-old male patient presented with recurrent ankle sprains on the right side. Elevated medial longitudinal arch is indicative of a cavus foot **(A)**M. Depressed 1^st^ metatarsal head can accompany plantar callus **(B)**. Observation of the medial border of the heel from the front, a positive peek-a-boo heel sign, is suggestive of heel varus **(C)**.

An elevated medial longitudinal arch in a weight bearing foot is indicative of a cavus foot (Fig. **1A**). Along with the high arch, prominent dorsolateral foot and the extensor digitorum brevis, claw toes, depression of 1^st^ metatarsal (Fig. **1B**), callosity under first or fifth metatarsal heads, thickening or callosities over the lateral border of the foot may be present in a cavus foot.

**Fig. (2):**
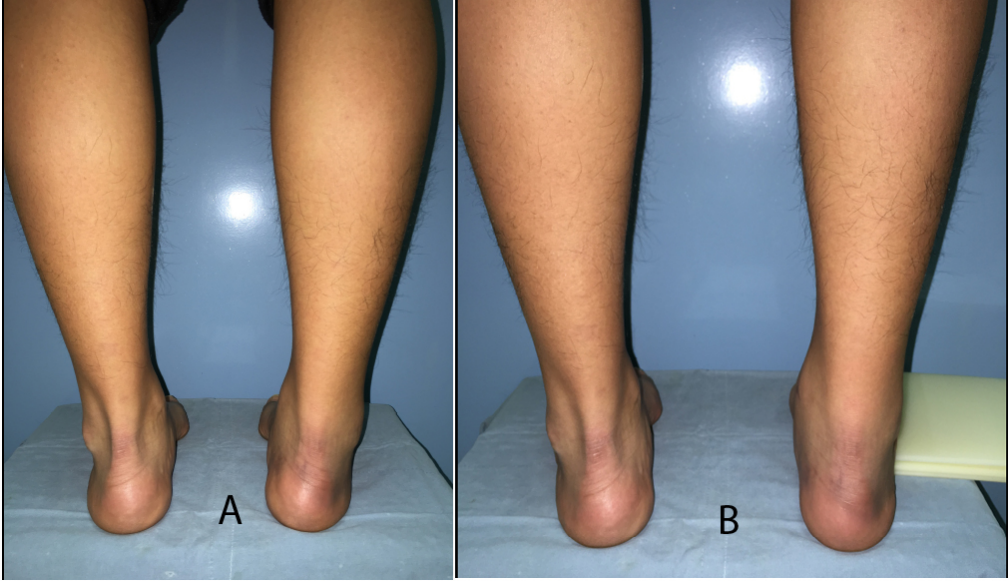
The same patient as in Fig. (**1**), seen from behind. Varus alignment of the right heel (A) is corrected by Coleman block test (B).

While severe cavus foot can be easily determined, a subtle cavus deformity requires careful inspection of the foot with high threshold of suspicion. A “peek-a-boo heel sign [13]” is gaining popularity as a sign of heel varus determined by mere observation from the front. With the patient standing with the feet at shoulder width apart and the medial borders of the hallux of both feet in parallel alignment, any appearance of the medial border of the heel is considered a sign of heel varus (Fig. **1C**). This sign is not present when there is valgus or neutral hindfoot position. Heel varus is then confirmed from the rear (Fig. **2A**) to rule out any possible false positive peek-a-boo sign due to a very large heel pad or severe metatarsus adductus with externally rotated lower extremities.

Once the heel varus is confirmed, the flexibility of the hindfoot varus should be checked using the Coleman block test [14]. A block is placed under the lateral side of the foot, allowing the first metatarsal bone to drop. If the hindfoot is flexible and the hindfoot varus is completely driven by the pronated forefoot, the heel varus will be corrected into slight valgus (Fig. **2B**). If the heel varus persists, further evaluation is required to investigate accompanying tarsal coalition, subtalar arthrosis, previous fracture, or muscular spasm.

A thorough neurological examination should be performed, paying special attention to the peroneus longus and brevis, as well as tibialis posterior and anterior muscles.

### 5 RADIOGRAPHIC SIGNS

**Fig. (3):**
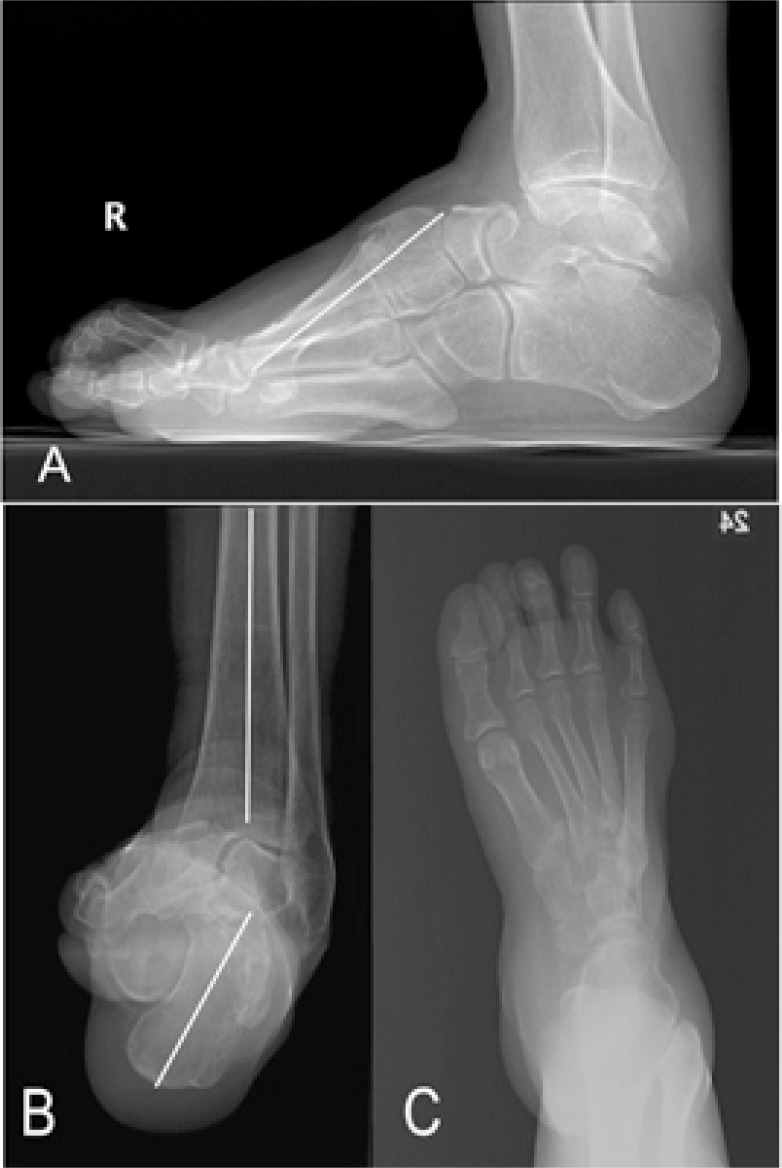
Radiographic findings of a patient with severe cavovarus foot. A standing lateral view **(A)** shows a plantar flexed 1^st^ ray, elevated navicular height, depressed 5^th^ metatarsal base, and claw toe deformities. Exact Meary angle can not be measured due to severe tilting of the talus. The posterior facet of the subtalar joint as well as the Chopart joints are clearly viewed in this lateral radiograph due to rotation of the hindfoot. The distal fibular is enlarged and posteriorly located. Secondary osteoarthritis of the ankle joint is noted. A heel-alignment view **(B)** confirms the degree of heel varus. From the dorsoplantar foot X-ray, the metatarsals are adducted and the 1^st^ metatarsal appears short due to plantar flexion.

The following radiographic features can help in considering the diagnosis of a cavus foot [4, 9, 15, 16] (Fig. **3**):

Increased calcaneal pitch (angle between a line along the undersurface of the calcaneus and the floor; normal is <30**°**).

Increased Meary angle (due to the plantar flexed first metatarsal, the angle between a line drawn along the axis of the first metatarsal and that of the talus is increased. Normal is 0 ± 5°)

Increased Hibbs angle (angle between a line through the axis of the calcaneus and the first metatarsal; normal is <45°; cavus is near 90°).

Increased navicular height

Posterior position of fibula (the fibula appears more posterior to the tibia than normal due to the varus hindfoot position and external rotation of the lower limb.).

Subtalar view (Due to the inversion of the hindfoot, the posterior facet of the subtalar joint is clearly visible in a lateral foot radiograph).

In order to correctly measure some of the angles mentioned above, true dorsoplantar and lateral weight-bearing foot radiographs are required. However, when the deformity is severe, the talus and calcaneus tilt into varus, making it impossible to draw a correct axis of the bone. Therefore, the reference values mentioned above are to be used as guidelines rather than definitive diagnostic criteria.

## 6 MANAGEMENT

### 6.1 Nonsurgical Treatment

Patients with milder symptoms associated with a cavus deformity can benefit from conservative treatment consisting of gastrocnemius muscle stretching exercise and specialized foot orthotics. The aim of applying an orthotic is to realign the hindfoot correctly to offload the lateral border of the foot. Therefore, an ideal orthotic for a subtle cavus foot should support the lateral hindfoot and midfoot with a wedge [17]. Medial arch support should be minimized since it can further tilt the foot in supination [9].

### 6.2 Surgical Reconstruction

When considering an operative treatment for a cavus foot, the goal is to obtain a stable plantigrade foot with preservation of joints if possible. In order to do that, one should recognize the muscle imbalance and understand the structural alterations in the foot. The foot will not be balanced with any uncorrected structural deformity and the deformity will recur if the foot is not balanced. So, for any cavus foot, one has to correct the muscle imbalance and correct any structural deformity.

Since every deformity is unique, there is no such thing as a standard protocol that can be applied universally. Instead, there is a list of many procedures and surgical options that we can choose from to optimally reconstruct each cavus foot (Table **2**).

#### 6.3 Soft Tissue Releases

Prolonged cavovarus deformities are almost always accompanied by tight heel cord and contracted medial and plantar soft tissues. Since it is impossible to correct structural deformity in the presence of contracted soft tissues, release of tight soft tissues must preceded to any other procedures.

Tight Achilles tendon can be lengthened by percutaneous triple hemisection, open Z-plasty, or by gastrocnemius recession. Silfverskiold test is useful to determine the components of Achilles tendon that require lengthening. Once the Achilles tendon is lengthened, more accurate assessment of the residual varus deformity becomes possible.

Plantar fascia should be completely released. It can be performed through a 3 cm long incision over its calcaneal insertion. In severe cavus feet, the abductor hallucis fascia may also require a release, which can be performed through the same incision. Care should be exerted not to injure the medial calcaneal branch of the tibial nerve as well as the nerve branch that inserts to the abductor hallucis muscle.

In severe cavovarus cases, additional release of posteromedial structures including flexor hallucis longus, flexor digitorum longus, posterior tibialis tendon can also be necessary. Release of the deltoid ligament can be performed if there is talar tilt in the ankle joint due to deltoid contracture.

#### 6.4 Bony Reconstruction

Correction of structural deformity requires either osteotomy or arthrodesis. If the hindfoot is flexible, determined by the positive Coleman block test, osteotomy can realign the cavovarus without scarifying the joint. Whenever possible, osteotomies are preferred over fusions. However, if the hindfoot varus is rigid, arthrodesis may be inevitable.

A positive Coleman block sign implies that the hindfoot varus is due to the plantarflexed 1^st^ ray and the hindfoot is flexible. Therefore, removing the deforming force by elevating the first ray must be performed. It can be achieved by a dorsiflexion osteotomy at the base of the first metatarsal. A dorsal wedge is removed at a point 10mm distally from the first tarsometatarsal joint. If the apex of the deformity is more proximal, arthrodesis of the 1^st^ tarsometatarsal joint or closing wedge osteotomy at the medial cuneiform can be considered.

**Fig. (4):**
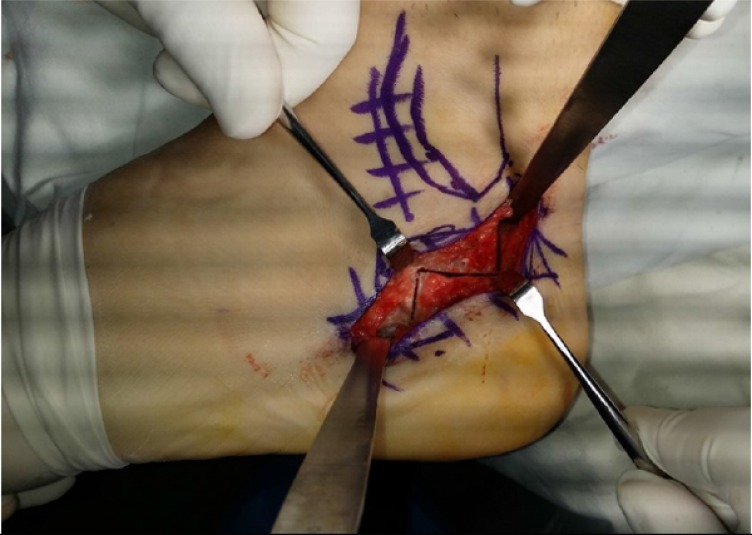
A Z osteotomy allows lateral translation of the posterior fragment. Removing small wedges from the anterior and posterior facets of the osteotomy can be added depending on the severity of the deformity. Care should be exerted not to injure the sural nerve.

If hindfoot varus is fully corrected with 1^st^ metatarsal osteotomy, then calcaneal osteotomy is not necessary. However, if there is residual varus after the dorsiflexion osteotomy, or if the Coleman block did not completely correct the hindfoot varus, a calcaneal osteotomy must be done. For a mild varus, a Dwyer closing wedge osteotomy [18] may be sufficient. For a greater amount of correction, lateralization osteotomy is necessary. An oblique osteotomy has the advantage of three-dimensional correction as the posterior fragment can be rotated, translated, and elevated. Rotation can be achieved with additional resection of a lateral based wedge. Elevation of the posterior fragment is helpful to decrease the calcaneal pitch. A Z osteotomy [7] is another powerful tool to correct the heel varus (Fig. **4**). The osteotomy primarily allows translation, but a little bit of rotation can be added by removing small wedges. Since the center of rotation is more anterior, the Z osteotomy allows a greater degree of correction compared to Dwyer osteotomy.

Salvaging joints wherever possible is beneficial because it allows more flexibility and shock absorption. However, rigid or severe cavus foot can only be reconstructed using arthrodesis. For a triple arthrodesis, the subtalar, talonavicular, and calcaneocuboid joints are denuded and fixed in a mild heel valgus position. The forefoot should be supinated through the Chopart joint [8]. When performing a triple arthrodesis, the cuboid can slide slightly beneath the calcaneus due to the natural shape of the calcaneocuboid joint. This causes a painful bony bump on weight bearing. To avoid this, it is useful to flat cut the calcaneocuboid joint with a saw. Excluding the calcaneocuboid joint in a triple fusion is also feasible since the calcaneocuboid joint is rarely arthritic. This is also beneficial because it reduces the potential of problematic nonunion of the calcaneocuboid joint.

#### 6.5 Muscle Balancing

If the deformity is originated from or related to any kind of muscle imbalance, a tendon transfer is always necessary. Without well-balanced muscle power, the deformity will recur and the correction will eventually fail.

Peroneus longus transfer to brevis is the most commonly performed tendon transfer. Since peroneus longus plantar flexes the 1^st^ metatarsal, removing this deforming force is essential in preventing the recurrence. It is also beneficial because the transferred peroneus longus tendon augments the peroneus brevis, which is frequently weakened or problematic. If the peroneus brevis is severely torn or degenerated, the pathologic portion should be repaired or excised before the transfer.

Posterior tibial tendon produces an unopposed pull in the presence of the peroneus brevis dysfunction. As a result, foot inversion and progressive contracture of the medial soft tissues will develop. Therefore, the goal of the posterior tibial tendon transfer is to weaken the deforming power and to strengthen the deficient function of the foot. The transferred posterior tibial tendon is inserted to one of the cuneiforms, where it functions as an ankle dorsiflexor.

In less severe deformities, the anterior tibial tendon can be transferred laterally to the middle cuneiform. Lateralizing the anterior tibial tendon reduces the supination vector while maintaining the dorsiflexion power. If strength of the anterior tibial muscle is maintained, an isolated transfer is performed. If the tendon is weak, augmentation with simultaneous transfer of the extensor digitorum longus can be considered [19].

Besides tendon transfers, repairing or augmenting the lateral ankle ligaments is frequently performed since lateral ankle instability is almost always accompanied in a cavus foot. Ligament repair with extensor retinaculum augmentation is the procedure of choice. Peroneus transfer to brevis also augments the lateral stability.

## CONCLUSION

Cavovarus deformity is closely related to various pathological conditions of the foot and the ankle. Even a small residual deformity or muscle imbalance can affect the treatment outcome. Therefore, thorough examination of the deformity and the deforming force should be performed before planning the treatment. Since every deformity is different, surgical procedures must be customized to each patient.

## Figures and Tables

**Table 1:** Clinical manifestations associated with cavus foot.

***Forefoot and Midfoot***
Metatarsalgia
Callus under 1^st^, 5^th^ metatarsal heads
Morton’s neuroma
Sesamoid problems (sesamoiditis, chondromalacia, avascular necrosis)
Stress fracture of metatarsal bones
Metatarsus adductus
Midfoot arthritis
***Ankle and hindfoot***
Plantar fasciitis
Achilles tendinitis
Chronic lateral ankle instability
Subtalar instability
Peroneal tendon problems (tear or split, rupture, tendinopathy)
Enlarged or posteriorly placed distal fibular
Recurrent dislocation of the peroneal tendons
Painful os peroneum syndrome
Painful Haglund deformity
Varus ankle arthritis

**Table 2:** List of surgical procedures for cavus foot.

***Correction of the structural deformity***
Soft tissue procedures
Achilles tendon lengthening
Plantar fascia release
Abductor hallucis fascia release
Deltoid ligament release
Lateral ankle ligament reconstruction
Osteotomies
First metatarsal dorsiflexion osteotomy
Midtarsal closing wedge osteotomy
Calcaneal valgizational osteotomy
Arthrodesis
Double or Triple fusion
First tarsometatarsal fusion
Naviculocuneiform arthrodesis
***Correction of dynamic muscle imbalance***
Tendon transfers
Peroneus longus tendon transfer to peroneus brevis
Posterior tibial tendon transfer to dorsum of foot
Anterior tibial tendon transfer to the middle of the foot
Extensor hallucis longus transfer to 1^st^ metatarsal (Jones procedure)
***Correction of the structural deformity***
***Correction of claw toes***
Soft tissue release
Resection arthroplasty
Proximal interphalangeal joint fusion
Girdlestone Taylor transfer

## References

[1] Irwin T.A., Anderson R.B., Davis W.H., Cohen B.E. (2010). Effect of ankle arthritis on clinical outcome of lateral ankle ligament reconstruction in cavovarus feet.. Foot Ankle Int..

[2] Manoli A., Graham B. (2005). The subtle cavus foot, “the underpronator”.. Foot Ankle Int..

[3] Sachithanandam V., Joseph B. (1995). The influence of footwear on the prevalence of flat foot. A survey of 1846 skeletally mature persons.. J. Bone Joint Surg. Br..

[4] Aminian A., Sangeorzan B.J. (2008). The anatomy of cavus foot deformity.. Foot Ankle Clin..

[5] Alexander I.J., Johnson K.A. (1989). Assessment and management of pes cavus in Charcot-Marie-tooth disease.. Clin. Orthop. Relat. Res..

[6] Nagai M.K., Chan G., Guille J.T., Kumar S.J., Scavina M., Mackenzie W.G. (2006). Prevalence of Charcot-Marie-Tooth disease in patients who have bilateral cavovarus feet.. J. Pediatr. Orthop..

[7] Ortiz C., Wagner E., Keller A. (2009). Cavovarus foot reconstruction.. Foot Ankle Clin..

[8] Manoli A., Beals T.C., Hansen S.T. (1997). Technical factors in hindfoot arthrodesis.. Instr. Course Lect..

[9] Abbasian A., Pomeroy G. (2013). The idiopathic cavus foot-not so subtle after all.. Foot Ankle Clin..

[10] Chilvers M., Manoli A. (2008). The subtle cavus foot and association with ankle instability and lateral foot overload.. Foot Ankle Clin..

[11] Tynan MC, Klenerman L, Helliwell TR, Edwards RH, Hayward M Investigation of muscle imbalance in the leg in symptomatic forefoot pes cavus: a multidisciplinary study.. Foot Ankle Jan..

[12] Helliwell T.R., Tynan M., Hayward M., Klenerman L., Whitehouse G., Edwards R.H. (1995). The pathology of the lower leg muscles in pure forefoot pes cavus.. Acta Neuropathol..

[13] Manoli A., Smith D.G., Hansen S.T. (1993). Scarred muscle excision for the treatment of established ischemic contracture of the lower extremity.. Clin. Orthop. Relat. Res..

[14] Coleman SS, Chesnut WJ A simple test for hindfoot flexibility in the cavovarus foot.. Clin Orthop Relat Res Jan.

[15] Solis G., Hennessy M.S., Saxby T.S. (2000). Pes cavus: A review.. Foot Ankle Surg..

[16] Maskill M.P., Maskill J.D., Pomeroy G.C. (2010). Surgical management and treatment algorithm for the subtle cavovarus foot.. Foot Ankle Int..

[17] LoPiccolo M., Chilvers M., Graham B., Manoli A. (2010). Effectiveness of the cavus foot orthosis.. J. Surg. Orthop. Adv..

[18] Dwyer F.C. (1959). Osteotomy of the calcaneum for pes cavus.. J. Bone Joint Surg. Br..

[19] Huber M. (2013). What is the role of tendon transfer in the cavus foot?. Foot Ankle Clin..

